# Assessment of the ecologically dependent post-zygotic isolation between *Anopheles coluzzii* and *Anopheles gambiae*

**DOI:** 10.1371/journal.pone.0240625

**Published:** 2020-10-29

**Authors:** Abdoulaye Niang, Simon Péguédwindé Sawadogo, Roch K. Dabiré, Frederic Tripet, Abdoulaye Diabaté

**Affiliations:** 1 Institut de Recherche en Sciences de la Santé, Bobo-Dioulasso, Burkina Faso; 2 Centre for Applied Entomology and Parasitology, School of Life Sciences, Keele University, Staffordshire, United Kingdom; Virginia Polytechnic Institute and State University, UNITED STATES

## Abstract

Within the *Anopheles gambiae* complex, the sibling species *An*. *coluzzii* and *An*. *gambiae* are undergoing sympatric speciation. These species are characterized by rare hybrids in most of their geographical distribution. A strong assortative mating mediated by spatial swarm segregation has been shown whereas no intrinsic post-zygotic barriers have been found in laboratory conditions. To test the role of the hybridisation in reproductive isolation in natural populations transplant experiment are therefore needed to establish the significance of post-zygotic barriers. Previous studies indicated that predation is one of the major forces driving ecological divergence between *An*. *gambiae* and *An*. *coluzzii*. Here we extended these studies to their hybrids. Parental species and their F1 hybrids from reciprocal crosses were generated by the forced-mating technique as follows: female *An*. *coluzzii* × male *An*. *coluzzii*; female *An*. *coluzzii* × male *An*. *gambiae*; female *An*. *gambiae* × male *An*. *coluzzii* and female *An*. *gambiae* × Male *An*. *gambiae*. First instar larvae of each group from the crossing (here after *An*. *coluzzii*, Hybrid COL/GAM, Hybrid GAM/COL and *An*. *gambiae*, respectively) were transplanted in a field experiment with predation effect. Emergence success, development time of larvae and body size of the newly emerging adults were estimated as fitness components and then compared between parental species and F1 hybrids in absence and in presence of predators. Our findings confirm that *An*. *coluzzii* had higher fitness than *An*. *gambiae* in presence of predators versus in absence of predators. Moreover, the fitness of the F1 hybrid COL/GAM whose female parent was *An*. *coluzzii* matched that of *An*. *coluzzii* while that of the F1 reciprocal hybrid GAM/COL was similar to *An*. *gambiae*.

## Introduction

Speciation involves the evolution of barriers to gene flow between diverging populations. Understanding speciation thus implies two major tasks: determining which reproductive barriers are involved in the reduction in gene flow between populations and, understanding which evolutionary forces produce them. Selection is known to be one of the most important biological processes in the formation of new species, that acts on individuals to let only the mostly fit offspring survive and reproduce to their full potential. Selection against hybrids, as an ecologically-dependent post-zygotic reproductive isolation occurs between species when hybrids are less efficient at exploiting parental environments and an intermediate environment is lacking [1, 2]. So, hybrids which suffer reduced fitness would not be well adapted to either parental environment, they would fall between niches. During the early phase of sympatric divergence, species may have been incompletely reproductively isolated populations, suggesting that ecotypes are excellent models for studying ecological speciation.

Within the *An*. *gambiae* complex, three sibling species *An*. *coluzzii* and *An*. *gambiae* [3] and *An*. *arabiensis* have the widest range of distribution and are responsible for the vast majority of malaria transmission in sub-Saharan Africa [4]. *An*. *coluzzii* and *An*. *gambiae* represent the most recent speciation event in the complex [5, 6] and are thought to be undergoing a process of speciation with gene flow [7–9]. Understanding the mechanisms of their pre-mating and post-zygotic reproductive isolation has important implications for vector control [10]. Assortative mating mediated by spatial swarm segregation between *An*. *coluzzii* and *An*. *gambiae* has been widely described as a pre-mating isolation barrier [11–16]. However, the current gene flow [17–19] and the extensive hybridization [8, 20–22] observed between *An*. *coluzzii* and *An*. *gambiae* could be the result of an imperfect assortative mating [23] notably due to the periodically break down in sympatric seasonal populations [13, 16].

While no intrinsic post-zygotic barrier has been found in laboratory conditions [24], previous studies have supported that *An*. *coluzzii* and *An*. *gambiae* larvae differ in their ability to exploit larval habitats in nature [25, 26]. *An*. *coluzzii* is better adapted to more permanent larval habitats mainly resulting from human activities, including rice fields, whereas *An*. *gambiae* grows up in temporary body waters mostly rain-dependent [21, 26–29] such as the edges of puddles. Many factors including desiccation, nutrients, competition, and predation are believed to affect strongly habitat selection of mosquito larvae. Several studies have demonstrated the role of predators in controlling mosquito population sizes in nature [30]. For example, an overall estimate of 94% mortality of mosquito larvae are due to predation [31]. The contrasted larval habitats of *An*. *coluzzii* and *An*. *gambiae* are associated with differences in the composition and abundance of aquatic mosquito predators [26]. Previously, predator pressure has been shown to be typically higher in areas of rice cultivation than in temporary puddles [32, 33], leading to the mosquito ecological segregation [26, 28, 34, 35]. Using transplantation of first instar larvae in a field experiment, [26] we estimated the development success of *An*. *coluzzii* and *An*. *gambiae* in both temporary puddles and permanent rice fields larval sites with a predation effect. The results have demonstrated that the two species are adapted to different types of larval habitats, leading to evolutionary implications for speciation. Unfortunately, until now, the information about how the parental environments of the two species directly affect the fitness of the hybrids in wild is lacking.

In the present study we tested a prediction of the ecological outcome concerning the fitness of hybrids between *An*. *coluzzii* and *An*. *gambiae* as one of the most important components of the extrinsic post-zygotic isolating mechanisms. Selection against hybrids will reinforce larval habitat preference of the parental species. In this scheme hybrids between *An*. *coluzzii* and *An*. *gambiae* would also have a lower mean fitness than either parental species. Accordingly, the high rate of hybridization between the two species observed along the Western coast of Africa [20, 22, 36] was typically unexpected due to the fact that a low fitness of the hybrids might be associated to their reduced survival and/or reproductive success. Thus, it suggests either a different mating behaviour or a loss selection pressure on hybrids in this part of Africa. Recently, studies have also demonstrated that asymmetric introgressions [17] and hybridization can be promoted by asymmetric over-dominance of *An*. *coluzzii* in sympatric seasonal populations of the two species [16]. So far most of the genomic data accumulated suggest that hybrids are selected against in most of the distribution of the two species [7, 8]. More recently we describe selection against F1 hybrids of *An*. *coluzzii* and *An*. *gambiae* between the mating stage and the more advance (larval and adult) stages [16], but no empirical study has specifically looked at it. Hybrid transplant experiments are needed to complement laboratory-based data explaining the rarity of *An*. *coluzzii* × *An*. *gambiae* hybrids [24]. This approach allows us to test the significance of ecologically-dependent post-zygotic barriers between these species. By means of transplant experiment, we estimated the development success in terms of mortality rates and time of larval stage of the parental species *An*. *coluzzii* and *An*. *gambiae* compared to their F1hybrids from reciprocal crosses. The either F1 hybrid types are so-called here COL/GAM or GAM/COL according to they are being from the cross mating of *An*. *coluzzii* females with *An*. *gambiae* males or from *An*. *gambiae* females with *An*. *coluzzii* males, respectively. The wing size, as a correlated index of body size which thought to be a primary component of fitness, was also measured and compared between the parental species and the F1 hybrids. Our results are of significant importance and lead to better understanding of the ongoing ecological speciation in these major malaria vectors within the *An*. *gambiae* complex.

## Results

A total of 5,400 first instar larvae were transplanted in 36 cages in the rice field of Bama in presence or absence of predators. The larvae were composed of *An*. *gambiae*, *An*. *coluzzii* and the two reciprocal F1 hybrids (i.e the hybrid COL/GAM and the hybrid GAM/COL).

### Developmental success

**Fig 1:**
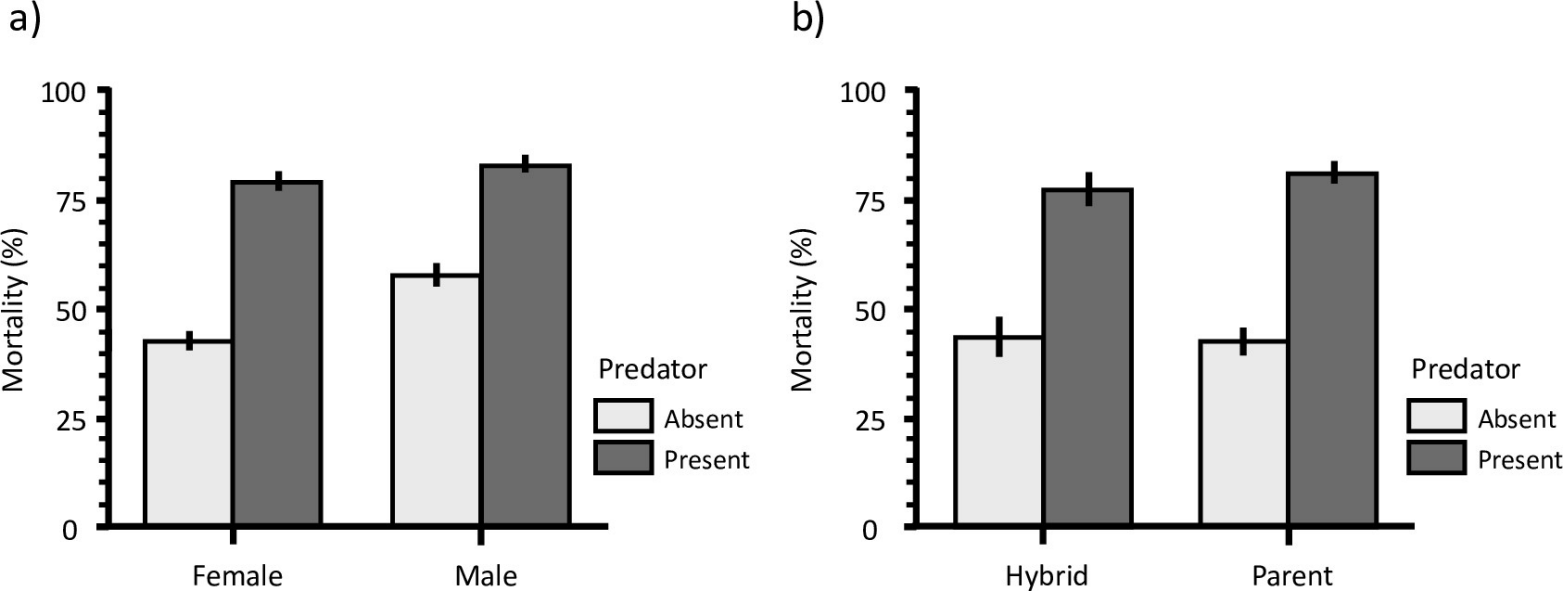
Comparison of the mortality rate (a) between males and females and, (b) between parents and hybrids in presence or absence of predators.

Overall 1,987 emergent adults were collected representing 36.8% of the total number of the initial transplanted larvae, with 63.2% of mortality regardless either in the presence or the absence of predators. The mortality rates of females recorded in presence of predator (78.1%) was significantly higher than in absence of predator (42%). The results were similar in males with a mortality rates of 82% in presence of predator and 50.7% in absence of predator (Fig 1A). The overall effects of the predator, replicates, cage, sex and their interactions on the mortality rate at emergence across all transplant cages were examined using the Logistic Regression Model (Table 1). Significant effects of the presence of predator, replicates, cage and sex were observed on the overall mortality rates of transplanted larvae (*P* < 0.001). As evidenced by their Chi-square contributions (Table 1), the presence of predator explained the highest proportion of variance, followed by those of cage and sex. Mortality was higher in cages with predators present and in males compared to females (Fig 1A).

**Table 1:** Logistic regression (effect likelihood ratio tests) of the effects of replicates, predator (presence or absence) cage (nested) and sex on mortality rates over all transplanted larvae.

Source	DF	L-R ChiSquare	P-value
Replicate	8	270.63	< 0.001*
Predator	1	852.78	< 0.001*
Cage [Predator]	26	503.64	< 0.001*
Sex	1	31.33	< 0.001*

As the hybrid males could not be identified by the PCR technique used in this study, the subsequent analysis was done on females only. Species identification was successfully performed on 1070 female specimens with only nine individuals failing to amplify. The mortality rate over both parental species (*An*. *gambiae* and *An*. *coluzzii*) was 80.3% in presence of predator and 41.9% without. Similarly, the overall mortality rate of hybrids from both reciprocal types of crosses was recorded at 76.6% and 43.1% in presence and absence of predator, respectively (Fig 1B).

**Fig 2:**
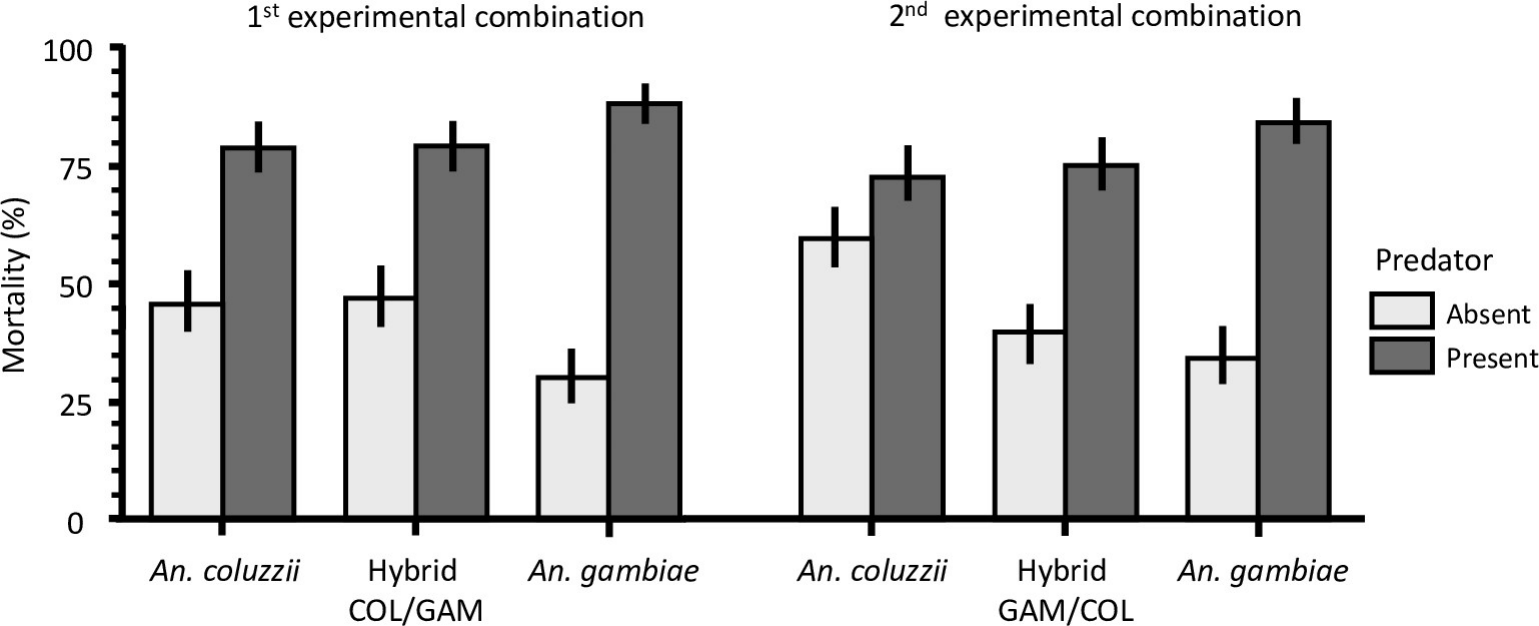
Comparison of the mortality rates of the pre-imaginal stages between the parental species *An*. *coluzzii*, *An*. *gambiae* and the Hybrid COL/GAM, and *An*. *coluzzii*, *An*. *gambiae* and the Hybrid GAM/COL in presence or absence of predators.

Next the mortality of the genotypes *An*. *coluzzii*, Hybrid COL/GAM and *An*. *gambiae* in the presence and absence of predator as well, within the first transplant experimental combination were analysed using Logistic Regression Model (Table 2, Fig 2). Predator presence strongly affected the survival of all genotypes to the adult stage (*P* < 0.001). There was no direct significant effect of genotype on mortality rates (*P* = 0.960), but a strong and significant interaction between genotypes and predation on mortality rates (*P* < 0.001) (Table 2). The mortality rate was significantly higher for *An*. *gambiae* (87.6%) compared to *An*. *coluzzii* (78.2%) and the Hybrid COL/GAM (78.7%) in presence of predator, while it was significantly less in absence of predators (Table 3, Fig 2) with 29.9% vs 45.3% for *An*. *coluzzii* and 46.7% for the Hybrid COL/GAM. In other words, *An*. *gambiae* performed better than either *An*. *coluzzii* or the hybrid COL/GAM in absence of predators, but both outcompeted the former one in presence of predator (Fig 2). No significant difference in the mortality rate of *An*. *coluzzii* and that of the hybrid COL/GAM was found (Table 3), they performed equally well in absence or presence of predators, suggesting that hybrid whose mothers are of *An*. *coluzzii* survived better the predator attacks. Differences in mortality rates of the genotypes *An*. *coluzzii*, Hybrid GAM/COL and *An*. *gambiae* in the presence and absence of predators (Table 3), as well as their performances were also analysed based on the similar model used above. Consistent with the data analyses in Fig 2, the Logistic Regression Model (Table 2) showed that predation has a significant effect on the mortality rates of the different genotypes (Log-likelihood Chi-square: *n* = 536, *df* = 2, *χ*^*2*^ *=* 33.03, *P* < 0.001). *An*. *coluzzii* exhibited a higher mortality rate (59.1%) than *An*. *gambiae* (33.9%) and the hybrid GAM/COL (39.5%) in absence of predators, but in presence of predators they were both poor competitors with 83.6% and 74.6% mortality for *An*. *gambiae* and the Hybrid GAM/COL respectively, vs 72% for *An*. *coluzzii* (Fig 2). No significant difference was found between *An*. *gambiae* and the hybrid GAM/COL (Table 3).

**Table 2:** Logistic regression (effect likelihood ratio tests) of the effects of predator (presence or absence) and genotype on mortality rates in the first experimental combination involving the parental species (*An*. *gambiae*, *An*. *coluzzii*) and F1 hybrid (COL/GAM) and the second experimental combination involving the parental species (*An*. *gambiae*, *An*. *coluzzii*) and F1 hybrid (GAM/COL).

	Source	DF	L-ratio	P-value
1^st^ experimental combination	Predator	1	251.55	< 0.001*
	Genotype	2	0.08	0.960
	Genotype*Predator	2	23.54	< 0.001*
2^nd^ experimental combination	Predator	1	153.80	< 0.001*
	Genotype	2	5.35	0.070
	Genotype*Predator	2	33.03	< 0.001*

**Table 3:** Pairwise group comparisons (log. likelihood odds ratios) of the effects of genotype on mortality rates in the first experimental combination involving the parental species (*An*. *gambiae*, *An*. *coluzzii*) and F1 hybrid (COL/GAM) and the second experimental combination involving the parental species (*An*. *gambiae*, *An*. *coluzzii*) and F1 hybrid (GAM/COL).

Experiment	Predators	Level 1	Level 2	Odds Ratio	P-value	Lw 95%	Up 95%
1^st^ experimental Combination	Present	Hybrid COL/GAM	*An*. *coluzzii*	1.05	0.777	0.728	1.529
		*An*. *gambiae*	*An*. *coluzzii*	0.51	0.001*	0.351	0.754
		*An*. *gambiae*	Hybrid COL/GAM	0.49	0.001*	0.333	0.715
	Absent	Hybrid COL/GAM	*An*. *coluzzii*	1.03	0.909	0.654	1.611
		*An*. *gambiae*	*An*. *coluzzii*	1.96	0.008*	1.188	3.285
		*An*. *gambiae*	Hybrid COL/GAM	1.91	0.011*	1.155	3.203
2^nd^ experimental combination	Present	Hybrid GAM/COL	*An*. *coluzzii*	1.14	0.538	0.751	1.731
		*An*. *gambiae*	*An*. *coluzzii*	1.97	0.003*	1.257	3.142
		*An*. *gambiae*	Hybrid GAM/COL	1.73	0.018*	1.097	2.768
	Absent	Hybrid GAM/COL	*An*. *coluzzii*	0.45	<0.001*	0.309	0.658
		*An*. *gambiae*	*An*. *coluzzii*	0.35	<0.001*	0.241	0.519
		*An*. *gambiae*	Hybrid GAM/COL	0.78	0.214	0.534	1.150

### Development time

**Fig 3:**
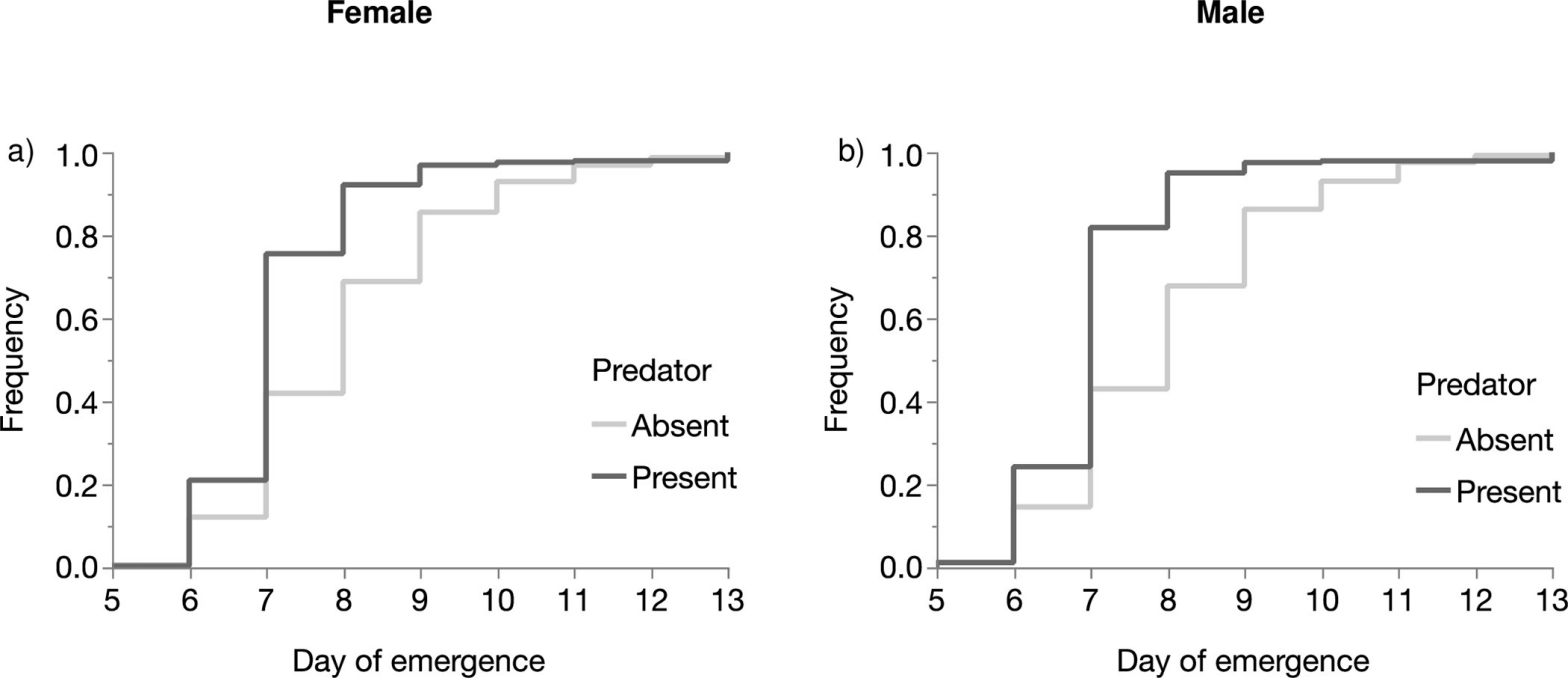
Comparison of the frequency of the emergent females (a) and males (b) through the development time of larvae reared in presence or absence of predators.

The overall development time varied from five to thirteen days. The variation in development time was significantly affected by the effects of replicate (*P* < 0.001), predator (*P* < 0.001) and sex (*P* = 0.047) (Table 4, Fig 3A and 3B). In females the mean numbers of day till emergence were 8.02 (95% CI = 7.48–8.61) and 7.20 (95% CI = 6.44–8.09) in absence and in presence of predators, respectively. In males the mean number of days till emergence was 7.97 (95% CI = 7.39–8.61) in absence of predator vs 7.06 (95% CI = 6.24–8.02) in presence of predators (Fig 3A and 3B).

**Table 4:** Proportional Hazard analysis (wald likelihood ratio tests) of the effects of transplantation, predator (presence or absence) and cage on the development time of larvae in transplant experiment involving the parental species (*An*. *gambiae*, *An*. *coluzzii*) and F1 hybrid (COL/GAM).

Source	DF	L-ratio	P-value
Transplantation	8	55.32	< 0.001*
Predator	1	10.25	0.001*
Cage ID [Predation]	27	251.20	< 0.001*
Sex	1	3.94	0.047*

**Fig 4:**
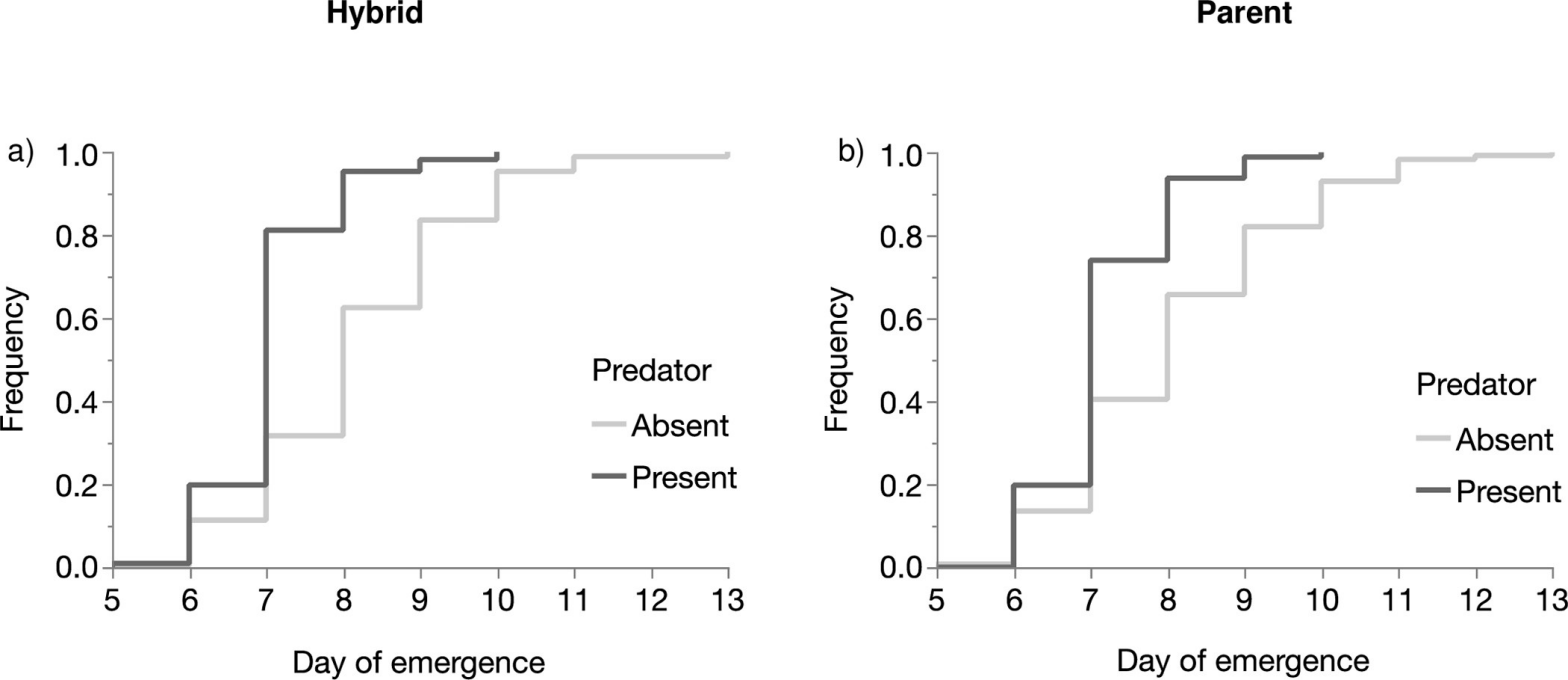
Comparison of the frequency of the emergent hybrids (a) and parents (b) through the development time of larvae reared in presence or absence of predators.

The subsequent analyses were performed for each experimental combination using the female genotype data. In the experimental combination composed of *An*. *coluzzii*, hybrid COL/GAM and *An*. *gambiae* genotypes, development time was strongly influenced by the predator presence (Proportional Hazard: *n* = 534, *df* = 1, *χ*^*2*^ *=* 97.7, *P* < 0.001) (Fig 4A and 4B). There was a significant effect of genotype on development time (Proportional Hazard: *n* = 534, *df* = 2, *χ*^*2*^ *=* 36.7, *P* = 0.001) (Table 5, Fig 4A and 4B); but no interaction between genotype (Hybrid and Parent) and predator presence (Proportional Hazard: *P >0*.*05)*.

**Table 5:** Proportional Hazard analysis (wald likelihood ratio tests) of the effects of predator (presence or absence) and genotype on the development time of larvae in the first experimental combination involving the parental species (*An*. *gambiae*, *An*. *coluzzii*) and F1 hybrid (COL/GAM) and the second experimental combination involving the parental species (*An*. *gambiae*, *An*. *coluzzii*) and F1 hybrid (GAM/COL).

Experiment	Source	DF	L-ratio	P-value
1^st^ experimental combination	Predator	7	97.73	< 0.001*
	Genotype	14	36.69	0.008*
2^nd^ experimental combination	Predator	8	100.76	< 0.001*
	Genotype	16	24.77	0.074

**Fig 5:**
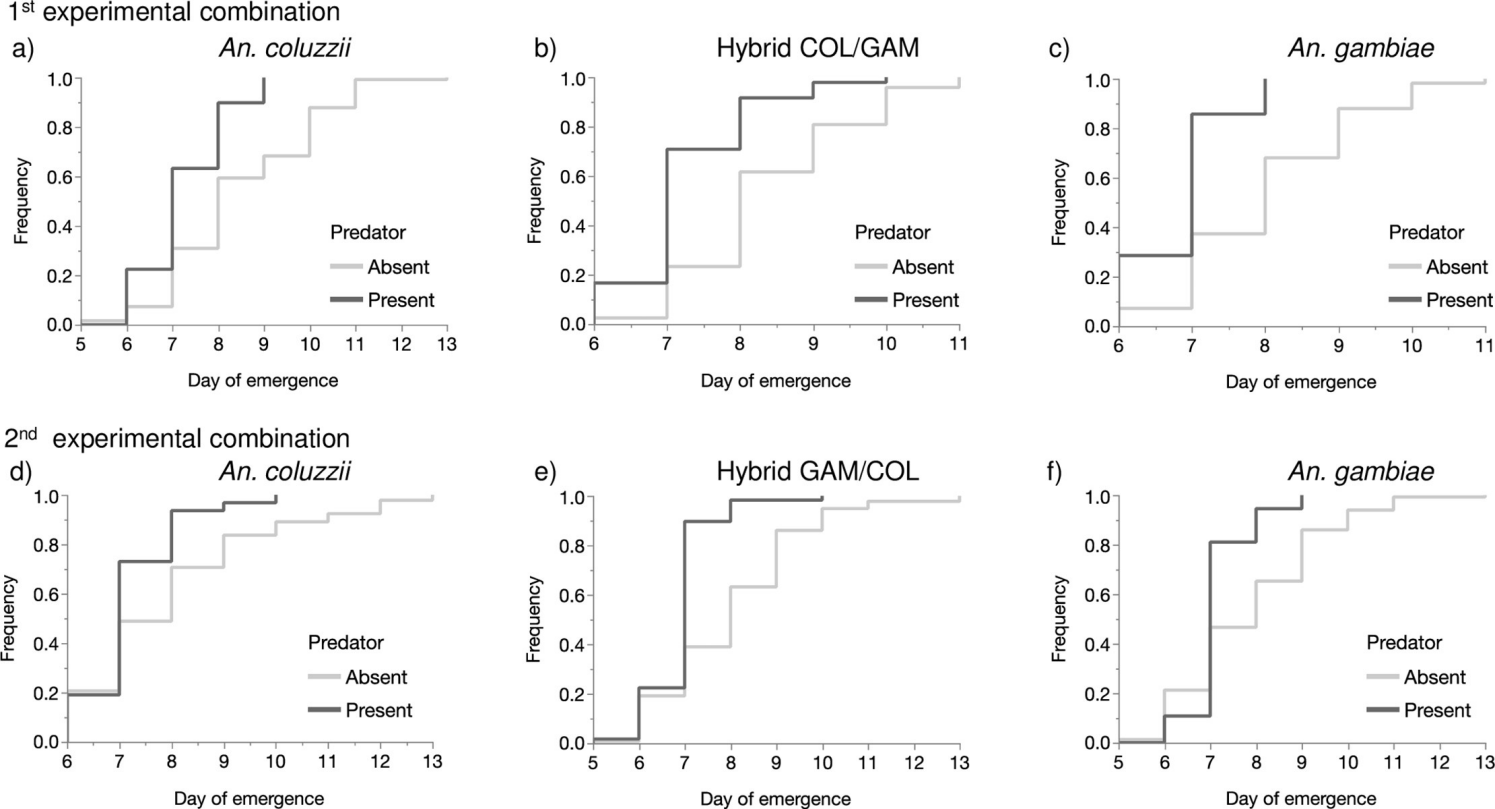
Comparison of the frequency of *An*. *coluzzii* (a) the Hybrid COL/GAM (b) and *An*. *gambiae* (c); *An*. *coluzzii* (d), the Hybrid GAM/COL (e) and *An*. *gambiae* (f) through the development time of larvae reared in presence or absence of predators.

In the first experimental combination the mean number of days till emergence was significantly higher in absence of predators with 8.46 (95% CI = 7.13–10.15) for *An*. *coluzzii*, 8.35 s (95% CI = 7.02–10.05) for the hybrid COL/GAM and 8.01 (95% CI = 6.91–9.36) for *An*. *gambiae*. When predators were present, these changed to 7.24 (95% CI = 5.54–9.72), 7.23 (95% CI = 5.52–9.73) and 6.86 (95% CI = 4.83–10.18) days for *An*. *coluzzii*, the hybrid COL/GAM and *An*. *gambiae*, respectively (Table 5, Fig 5A–5C).

In the second experimental combination, there was again a very significant effect of predator presence on the mean number of days of emergence (Proportional Hazard: *n* = 534, *df* = 8, *χ*^*2*^ *=* 100.8, *P* < 0.001). Developmental times were significantly shorter in absence of predators with 7.97 (95% CI = 6.54–9.84) for *An*. *coluzzii*, 8.01 (95% CI = 6.81–9.53) for the Hybrid GAM/COL and 7.87 (95% CI = 6.73–9.27) for *An*. *gambiae* than in presence of predator with 7.17 (95% CI = 5.66–9.28), 6.89 (95% CI = 5.39–9.02) and 7.13 (95% CI = 5.25–10.03) for *An*. *coluzzii*, the hybrid GAM/COL and *An*. *gambiae* respectively (*P* < 0.008) (Fig 5D–5F) (Table 5). However, there was not significant effect of genotypes on development time (*n* = 534, *df* = 16, *χ*^*2*^ *=* 24.8, *P* = 0.074) and no significant interaction (Proportional Hazard: *P >* 0.05).

### Adult body size

**Fig 6:**
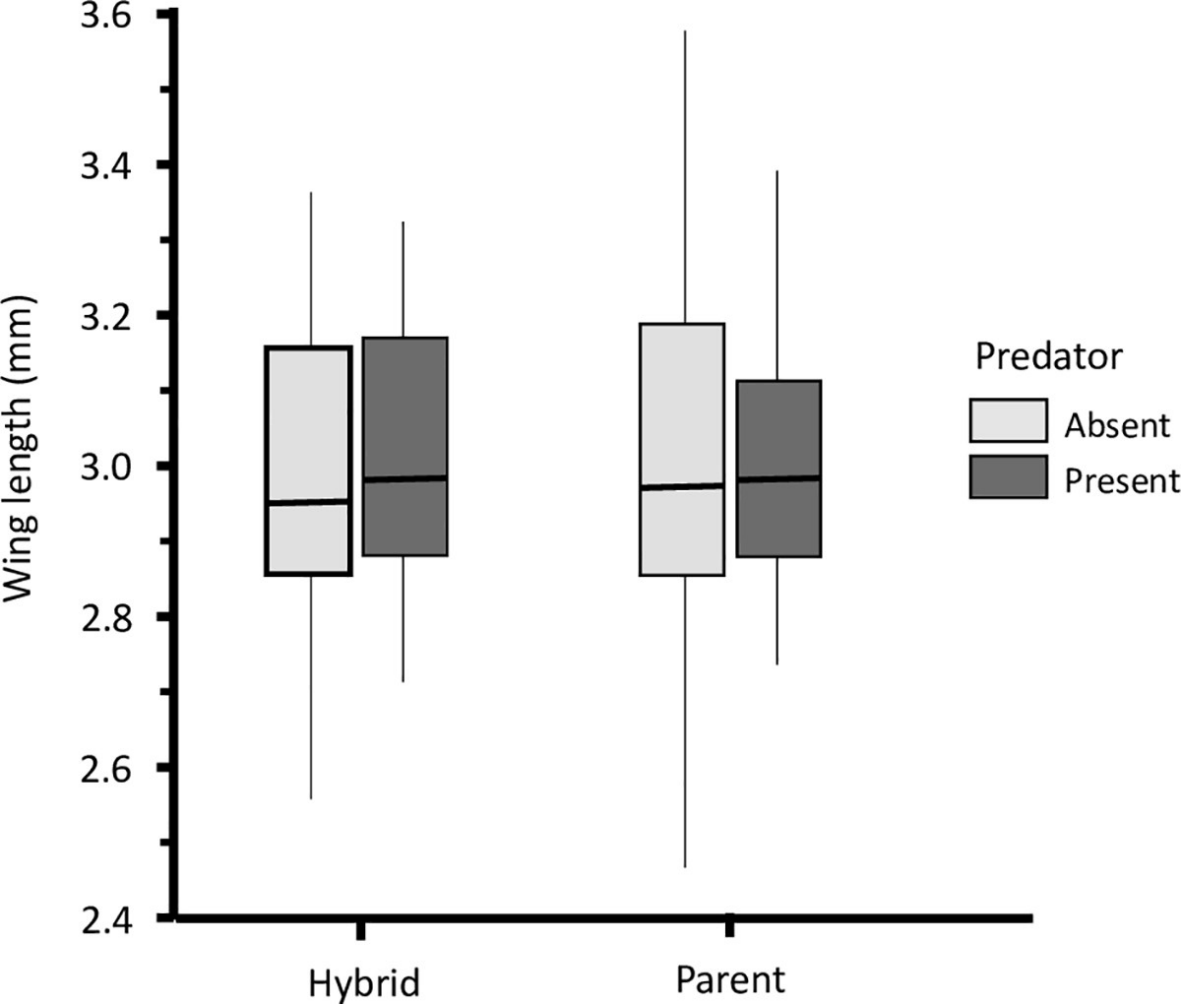
Comparison of the wing length between parents and hybrids in present or absence of predators between in predator present cages and predator free cages.

Adult female wing size averaged 3.00 mm and ranged from 2.46 mm to 3.57 mm. Across both experimental combinations combined, females were significantly larger in the hybrid COL/GAM experimental combinations than in the hybrid GAM/COL combinations (General linear model: *n* = 135, *df* = 7, *χ*^*2*^ *=* 19.41, *P* < 0.001). Females were also significantly larger when predators were present (GLM: *n* = 135, *df* = 1, *χ*^*2*^ *=* 6.47, *P* = 0.012). There was no overall difference in the wing size of individuals from the parental species and hybrids (GLM: *n* = 135, *df* = 1, *χ*^*2*^ *=* 0.008, *P* = 0.928) (Fig 6). The interaction between type of individuals (parent or hybrid) and predator (present or absent) was not significant (GLM: *n* = 135, *df* = 1, *χ*^*2*^ *=* 0.20, *P* = 0.653).

**Fig 7:**
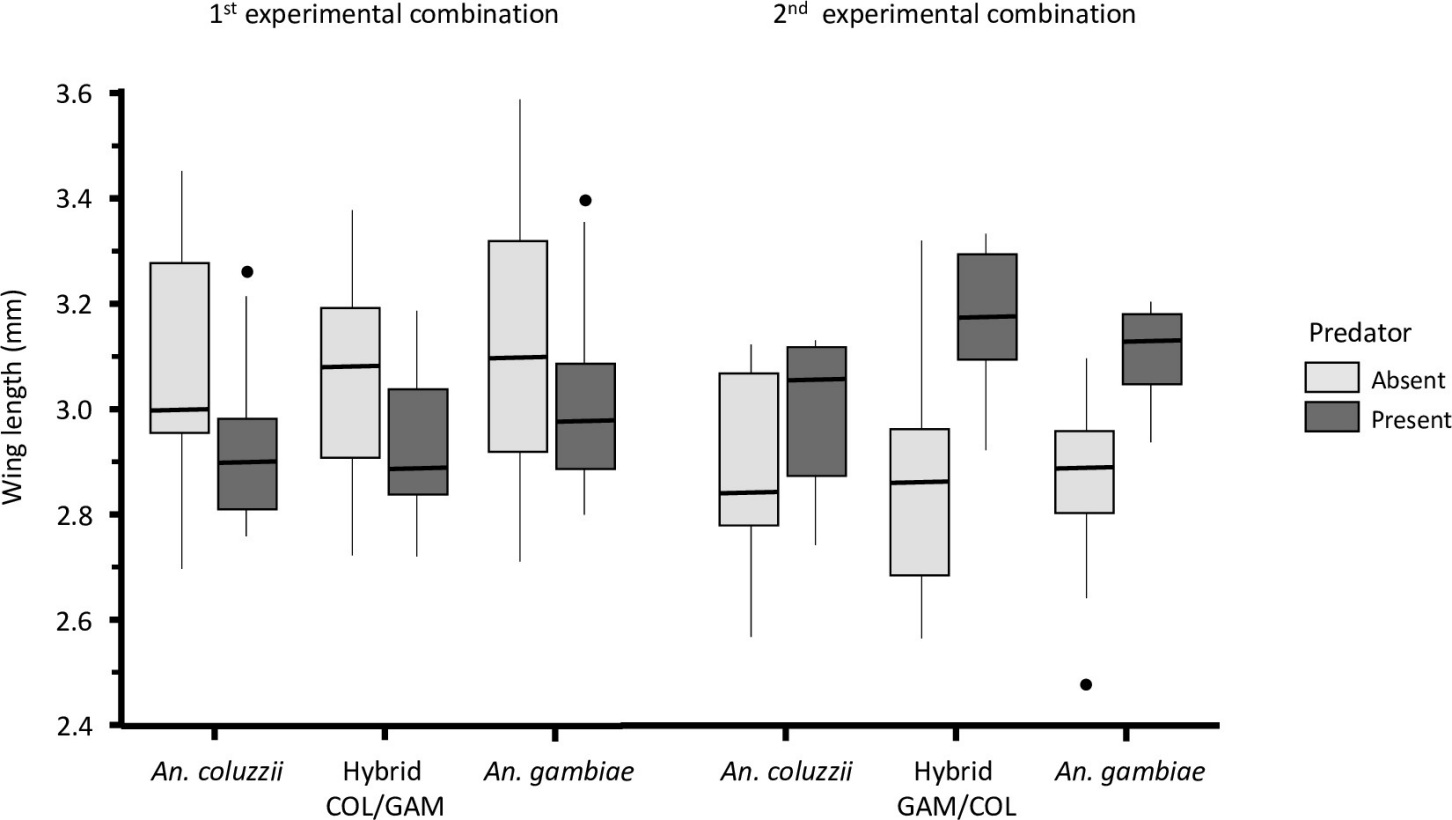
Comparison of the wing length between *An*. *coluzzii*, the Hybrid COL/GAM and *An*. *gambiae* and, *An*. *coluzzii*, the Hybrid GAM/COL and *An*. *gambiae* in present or absence of predators.

Conducting separate analyses for the hybrids COL/GAM and GAM/COL experimental combinations showed that in the first experimental combination neither the presence of predator nor female genotype affected wing size (GLM: predator, *n* = 84, *χ*^*2*^ *=* 0.05, *P* = 0.826; female genotype, *df* = 2, *χ*^*2*^ *=* 2.32, *P* = 0.105) (Fig 7). In the second experimental combination, a strong significant effect of predator was found on the wing size (GLM: *n* = 51, *df* = 1, *χ*^*2*^ *=* 27.03, *P* < 0.001) (Fig 7). Genotype did not have a significant impact on wing size (GLM: *df* = 2, *χ*^*2*^ *=* 1.11, *P* = 0.339).

## Discussion

In the present study, we investigated ecologically dependent post-zygotic barriers thought to have played an important role in the ecological speciation of the recently diverged species *An*. *coluzzii* and *An*. *gambiae*. First instar larvae of the two parental species *An*. *coluzzii* and *An*. *gambiae* and their F1 hybrids from bidirectional reciprocal crosses of the two species were transplanted in a rice field area and randomly assigned to experimental enclosures with or without predators. For the first time, life history components such as survivorship, development time and body size were compared between the parental species and their reciprocal F1 hybrids in relation to predation.

In this study, predator presence was associated with significant mortality and a major determinant of emergence success. The mortality rate was also significantly higher for *An*. *gambiae* than it was for *An*. *coluzzii* in presence of predator, but the reverse was true in absence of predators, thereby confirming that diverging responses to predators and the resulting *genotype*-*environment* interaction (*G×E*) may play an important role in niche partitioning and speciation. Most interestingly, the mortality rate of the hybrid COL/GAM (whose mothers were *An*. *coluzzii*) did not differ from that of *An*. *coluzzii*, while the hybrids GAM/COL (whose mothers were *An*. *gambiae*) performed like *An*. *gambiae*. In the first experimental combination event though *An*. *coluzzii* and the Hybrid COL/GAM are better competitor than *An*. *gambiae*, they would be disadvantage by the number of individuals which was initially two folders higher than those of An. gambiae. However, in the second experimental combination in addition that *An*. *coluzzii* had a better strategy in presence of predator, the number of individuals of *An*. *coluzzii* was to folders smaller than those of *An*. *gambiae* and the Hybrid GAM/COL combined. This could provide double advantages to *An*. *coluzzii* which developed better in the second experimental combination compared to the first experimental combination and the observed difference of the mortality rates. Predation is responsible for a high proportion of larval mortality in natural populations sometimes reaching over 90% [30, 37]. Estimations of the aquatic mortality due to the predation varied also from 2% to 96% in a semi-field experiment in Kenya [38]. In a previous larval transplantation experiment of *An*. *gambiae* and *An*. *coluzzii*, the latter survived better than *An*. *gambiae* in presence of predators [26], which further studies attributed to better behavioural predation avoidance [29, 34, 35].

The results of this study do not support the hypothesis that a lower fitness of F1 hybrids larval stages might contribute to extrinsic post-zygotic reproductive barriers between the sister species. Instead, F1 hybrids from both reciprocal crosses had similar reaction norms in response to predator species to that of their female parent species. This suggest that predatory avoidance behaviour was inherited from mothers possibly through loci in the genome acting in a maternal dominance pattern. Maternal effects have been widely recognized as important factors which influence offspring phenotype in insects [39–41] and they have been linked to reproductive pre and post-zygotic isolation processes in the Diptera, Lepidoptera and Orthoptera [42, 43]. In hybridization studies of three closely-related species of *Chorthippus* grasshoppers, results revealed contrasted modes of inheritance for female courtship song preferences [42, 44, 45]. Strong maternal effects were found in *Chorthippus biguttulus*/*mollis* hybrid females [46, 47] even though, in this context further additional backcrossing studies should aim to better understand the genetic and epigenetic processes involved.

Results of this study have shown a higher overall larval mortality in males resulting in an adult sex-ratio biased towards females. The number of transplanted larvae in this scheme was supposed to be comprised of both sexes, assuming a 1:1 sex ratio at hatching, though this is a method frequently used to quantify males and females first instar larvae in mosquitoes [26, 48]. Previous studies revealed that male and female larval survival in mosquitoes can be differentially affected by many factors that are dependent on the species and environmental conditions. Survival may be equal for both sexes [49] or stronger for female [50] or for male [51].

Our data on the developmental time shows that overall larvae of both sexes developed faster in presence of predators. Parental species and the two reciprocal hybrids had similar development time in absence of predator with no significant effect of the genotype noted in either experimental combination. Specifically, both parental species and hybrids developed faster in presence of predator. Increased developmental rate is likely due to the higher mortality observed in presence of predators which resulted in lower densities hence decreased resource competition. From an evolutionary point of view, and provided that larvae have access to sufficient food, it is possible that larvae accelerate their development in presence of aquatic predators as an adaptation to avoid predation [52]. Our data on body size does not support the hypothesis that this acceleration was traded against phenotypic quality (see below). In two studies, exposing *An*. *coluzzii* larvae to the presence of backswimmers or fishes induced stress which reduces larval survivorship but also increased development time [53, 54]. An obvious difference between the results presented here and those reported from laboratory studies is that in the latter, larvae might have had less opportunity to increase food intake whilst the larvae in our field cages had access to natural food sources.

Compatible with the idea that predation reduced larval density and food competition, overall, larvae reared with predators also achieved larger body sizes than those without predators. Reduced larval density resulting from predation [30, 37] leads to a shorter development time of mosquito larvae as shown above in this study and those that have been previously documented [52], suggesting larger emergent adults in presence of predators. In the current study, result show no overall significant effect of the predator presence when we combine the two experiments involving the two reciprocal hybrids in our analysis. Taken separately, predators effects on females body size were strongly significant (Fig 7), truly indicating larger females in presence of predators. Female size was found to be similar for the three genotypes of *An*. *coluzzii*, reciprocal hybrids and *An*. *gambiae* in presence of predators as well as in absence of predators. Nevertheless, the trend of intermediate size of the hybrid COL/GAM and those of larger size in the hybrid GAM/COL compared to the parental species in presence of predator suggest a strong relationship between the body size and the development time. Results from this study are consistent with findings in many other studies which have reported that the larval development and the body sizes of adult mosquitoes are larval food and density-dependent, thereby affecting fitness components [55–57]. In this experiment, mosquitoes may have access to more food in the predator presence, which may have led to a larger size relative to the absence of predator. However, mosquito larvae, including those of *Anopheles* species are known to adopt a low-risk behavior in habitats where conspicuous predators are present, spending more of their time resting and less time feeding [28, 29, 34, 58]. Such plasticity in behavior and life-history traits reflects changes in foraging cost/benefit in response to predators, a phenomenon that is widespread in invertebrate prey-predator systems [53, 54, 59–61].

## Conclusion

In replicated transplantation experiments of *An*. *gambiae*, *An*. *coluzzii* and reciprocal hybrids, predator presence was shown to strongly negatively impact larval survivorship and its effect was stronger in males and genotype dependent. In presence of predators, the parental species *An*. *coluzzii* and the COL/GAM hybrid survived better than *An*. *gambiae* and the reciprocal hybrid GAM/COL. These results highlight a strong maternal effect on larval responses to predators that is compatible with male X chromosome silencing or possibly other genetic or epigenetic processes. A consequence of this maternal effect, there was no measurable fitness cost on F_1_ hybrids as their survival and development matched that of their mother. The negative impact of predation on larval density led to larger body size of surviving females, and an unexpected shortening of their developmental time. Thus, these findings show how predation pressure experienced by larvae have implication on life-history traits of *An*. *coluzzii*, *An*. *gambiae* and their hybrids and they contribute to our understanding of reproductive barriers between sibling species. Future experiments focusing on backcross hybrids may help identify whether hybrid fitness costs play a role in other important phenotypes beyond the larval stage and F1 generation. These future investigations will help determine how patterns of gene flow between *An*. *coluzzii* and *An*. *gambiae* impact hybrid fitness, and will help us understand how hybrid fitness will impact the efficacy of genetic vector control strategies for vector control and eradication.

## Material and methods

### Mosquito collection and laboratory rearing

Indoor resting gravid females belonging to *Anopheles* genus were collected using mouth aspirators during the rainy season from July to September 2012 in VK5 (4° 25’ 00”W,11° 24’ N) and Soumousso (4° 02’45” W; 11° 00’ 46” N), in western Burkina Faso. Mosquitoes were transported to the insectary at the *Institut de Recherche en Sciences de la Santé (IRSS)* in Bobo-Dioulasso and maintained under standard conditions (28 ± 1°C, 80 ± 10% RH and 12–12 L:D). Thereafter, they were provided with 5% glucose solution for two nights. On the third day after collection, each female was placed in an individual cup for egg-laying. Females that laid eggs were killed and placed individually in tubes for identification as belonging to *An*. *gambiae* s.l. using the morphological key described by Gillies & De Meillon [62] and, then identified to species level using the PCR diagnostic [63, 64]. Newly hatched larvae from families identified as *An*. *coluzzii* or *An*. *gambiae* were pooled by species and reared in similar conditions. When they reached the pupal stage, they were sexed and placed in separate female and male cages. Following emergence, virgin adults were kept in these cages for three to five days supplied with 5% glucose solution.

### Forced mating procedure

Females were fed with blood of rabbits using an artificial blood feeding system and force mated on the fourth day. Females were briefly anesthetized with ether and laid on white filter paper, ventral side up. Males were fixed on insect pins by the thorax. The legs and heads of males were removed before they were presented to the females. Males were stimulated by gentle stroking of the genitalia with the female genitalia to induce copulation. Copulated females were kept for two days with 5% glucose solution and blood fed again. Thereafter, they were placed in individual cups for oviposition. To generate parental species and their reciprocal hybrids forced mating was done as follow: female *An*. *coluzzii* × male *An*. *coluzzii*; female *An*. *coluzzii* × male *An*. *gambiae*; female *An*. *gambiae* × male *An*. *coluzzii* and female *An*. *gambiae* × male *An*. *gambiae*.

### Transplant experiment

Parental species *An*. *coluzzii* and *An*. *gambiae* and, their F1 hybrids from reciprocal crosses COL/GAM and GAM/COL were transplanted in permanent larvae habitats of rice fields in Bama with or without predation effect by using transplant cages as described in Diabaté et al, 2008. Cylindrical cages of 70 cm diameter and 80 cm height were made of metal frame fitted from the bottom to the middle with a cloth. The elliptic pores of the cloth with a mean length of 0.12 mm and mean width of 0.08 mm contain the larvae but allow exchange of water, small particles, and microorganisms. The cage was covered with a mosquito net from the middle to the top, to avoid that adult mosquitoes and other invertebrates enter or exit the cage. The cage was fixed to the ground and secured by three stakes. First-instar larvae (L1) were randomly chosen on the day of hatch and used for establishing experimental cohorts of 150 larvae each. A precedent study [24] has showed no sex distortion in mean sex ratio at emergence in *An*. *coluzzii*, *An*. *gambiae* and their reciprocal hybrids. One experimental combination included fifty larvae from each of the three lineages *An*. *coluzzii*, F1 Hybrid COL/GAM and *An*. *gambiae*. Similarly, 150 larvae from each of the three following lineages *An*. *coluzzii*, F1 Hybrid GAM/COL and *An*. *gambiae* were also pooled into the second experimental combination. Both cohorts of larvae were reared in the absence and presence of two individuals of backswimmer, *Anisops jaczewskii* Hutchinson 1928 (Hemiptera: Notonectidae). This species was previously found to be the most abundant predator of Anopheles larvae widely spread throughout the rice field area of Bama [26]. It has been used in several studies for estimating the predation effect on mosquito larvae [26, 28, 29, 34]. Late 4th and 5th instars juveniles of backswimmer were collected and kept in the insectary and starved for 48 hours prior to the experiments. Experiments in which one or both backswimmers were died or escaped were removed from the analysis. Emerging adults from each cage were collected daily, counted and placed in micro tubes containing 70% alcohol for further identification by PCR diagnostic and wing measurements.

### Parental species and hybrid identification

DNA extraction from a single leg was used for identifying emergent females to species level by PCR [63]. Because hybrid males are hemizygous for the X-chromosome they cannot be identified using the species molecular diagnostics based on polymorphisms in the rDNA region of that chromosome [63, 64]. Consequently, only emerging females were used for the subsequent analysis.

### Wing size measurement

Because adult body size in anopheline mosquitoes depends strongly on environmental conditions during larval development [65], processes affecting larvae are directly linked to the final adult size [66]. Moreover, body size and wing length were found positively correlated in anopheline mosquitoes [67]. Wings from newly emergent adults were dissected, mounted dry on microscope slides, and photographed using a Leica EZ4 D (Leica Microsystems, Suisse) microscope. The wing length was measured using the software Image J1.41.0 (Wayne Rasband National Institute of Health, USA), including a random number of mosquitoes for which the right wing is intact. The length was measured as described previously from the posterior anal cell margin to the tip of radial vein 3 excluding fringe scales. [68]. To reduce confounding effects, only wings from the right side of the mosquitoes were selected and measurements were conducted by the same person.

### Ethical considerations

A visit to the collection sites was organised prior to any activity in order to meet the administrative and customary authorities of the VK5 and Soumousso villages to explain the objectives of the study and obtain their consent to participate in the study. Participation is completely voluntary and consists of giving access to the interior of one's home for the collection of female mosquitoes or to one's field for transplanting experiments in the rice fields in Bama. Any information on the identity of the participants will not be divulged in any way by the research team, the project coordinators or any other person involved in the study. All names were coded in order to remain anonymous. The owner decides whether or not to participate. He will not be penalized in any way if he refuses. Likewise, he can, if he wishes, freely decide to stop participating in this study. We have obtained ethical consent for the study in general from the local village councils and leaders, as well as specific consent from the owners of the houses or the rice fields.

### Statistical analyses

The results were analyzed to evaluate if there is a statistically significant difference between the predator-present cages and the predator-free cages, in terms of mortality at emergence, development time of larvae and size of newly emerging adults. Statistical analyses were carried out using the software JMP 10 (SAS Institute, Inc) through Logistic Regression Modelling.

## Supporting information

S1 Data (CSV)

S2 Data (CSV)

S3 Data (CSV)

S4 Data (CSV)

S5 Data (CSV)
